# Rituximab Treatment in a Patient with Active Graves’ Orbitopathy and Psoriasis

**DOI:** 10.4274/tjo.26780

**Published:** 2017-01-17

**Authors:** Tülay Şimşek, Nilgün Yıldırım, Belgin Efe, Nur Kebapçı

**Affiliations:** 1 Eskişehir Osmangazi University Faculty of Medicine, Department of Ophthalmology, Eskişehir, Turkey; 2 Eskişehir Osmangazi University Faculty of Medicine, Department of Internal Medicine, Division of Endocrinology, Eskişehir, Turkey

**Keywords:** Graves’ orbitopathy, Rituximab, Psoriasis

## Abstract

Management of Graves’ orbitopathy remains an important therapeutic challenge. Current therapeutic modalities are unsatisfactory in about one third of patients. Rituximab is a monoclonal antibody against CD20 antigen that is expressed in mature and immature B cells. Early experience with rituximab suggests that it is a promising alternative therapy for Graves’ orbitopathy. Here we report a case of a 49-year-old woman with Graves’ orbitopathy and psoriasis. The patient received 2 infusions of 1 g rituximab 2 weeks apart. Although there was improvement in inflammatory signs of the disease, proptosis did not change after the treatment.

## INTRODUCTION

Graves’ disease (GD) is an autoimmune disease that affects multiple systems including the thyroid, orbits and skin.^1^ Graves’ orbitopathy (GO) is the most common (in 25-50% of GD patients) and serious clinical manifestation of extrathyroidal GD.^2^ It has been shown that hyperthyroidism in GD results from the stimulation of thyroid stimulating hormone (TSH) receptors located on thyrocytes by immunoglobulin G, which is continuously produced by B cells. Although the pathogenesis of GO has not been fully elucidated, it is believed to be related to immunologic cross-activity between thyroid and orbital tissue antigens.^3^ Orbital fibroblasts are the primary cellular target of this autoimmunity. Autoantibodies produced in GD activate orbital fibroblasts, which stimulates the release of T cell cytokines and the subsequent synthesis of extracellular matrix components. TSH receptor (TSHR) autoantibodies as well as insulin-like growth factor 1 receptor (IGF-1R) autoantibodies are also present in GD, and they stimulate the production of T cell chemoattractants. B lymphocytes are reportedly responsible for the production of TSHR and IGF-1R antibodies. IGF-1R has been found on the surface of both T and B lymphocytes.^4^ Considering these data, it is understandable that there are many mechanisms responsible for GO pathogenesis and thus the search for the most appropriate treatments for this disease is still ongoing.

Immunomodulatory therapy has recently emerged as a treatment option for patients with mild to moderate active GO. Rituximab is a monoclonal antibody against the transmembrane protein CD20 found in both mature and immature B cells. CD20 antigen enables B cell activation and differentiation.^5^ There are several studies regarding the use of intravenous (IV) rituximab therapy in GO patients.^6,7,8^ These studies report a rapid reduction in GO activity score following rituximab infusion, no relapse for over 18 months and no serious drug-related side effects.^6,7^ With this report, we aimed to determine the efficacy and safety of IV rituximab therapy in a patient with GO and psoriasis and to evaluate therapeutic approaches in such cases.

## CASE REPORT

**Figure 1:**
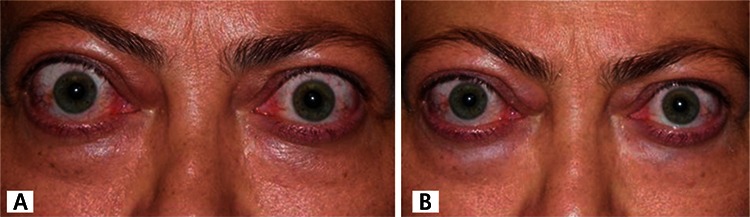
A) Pretreatment photograph showing bilateral proptosis which is more pronounced on the right, lid retraction, lid hyperemia and edema, conjunctival hyperemia, and right caruncular edema; B) posttreatment photograph showing bilateral improvement in lid edema, hyperemia, lid retraction, and conjunctival hyperemia

**Figure 2:**
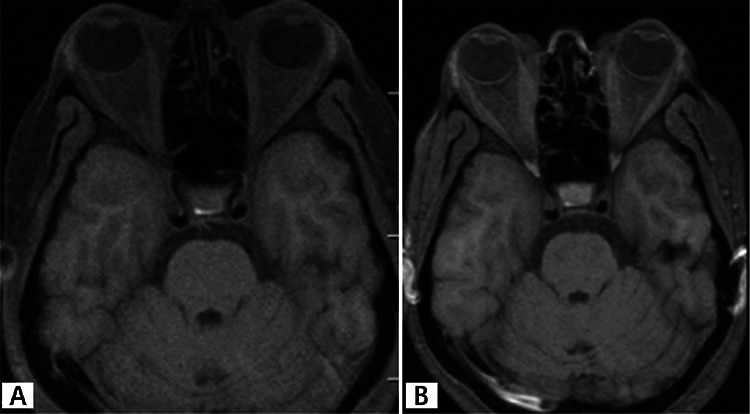
Orbital magnetic resonance imaging showing A) pretreatment fusiform thickening of the extraocular muscles and B) posttreatment regression of the extraocular muscle thickening

**Figure 3:**
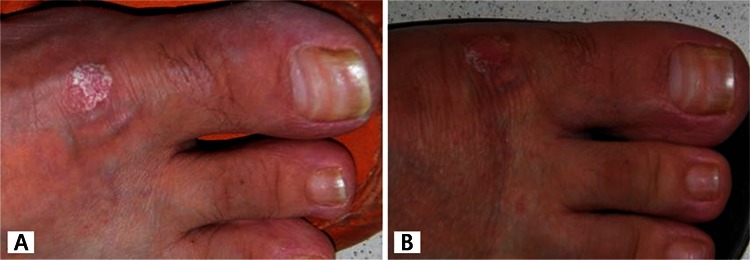
A) Pretreatment photograph showing a light-colored psoriatic lesion on the top of the foot; B) posttreatment photograph showing regression of the psoriatic lesion

A 49-year-old female patient presented with hyperemia, pain, proptosis, and blurred vision in both eyes. The patient had a 2-year history of hyperthyroidism and 35-year history of psoriasis. It was learned that she had radioactive iodine therapy 1.5 years earlier and her ocular symptoms had started about 1 year after this treatment. The patient also reported having stomach discomfort and a smoking habit. She was diagnosed with active GO. Rituximab was chosen for treatment because she was already taking adalimumab for psoriasis and was contraindicated for steroid use. Full ophthalmologic examination, visual field and visual evoked potential (VEP) tests were done prior to treatment and at 2 weeks, 1 and 2 months, and 1 year after treatment. GO was assessed using Hertel measurement, Hess screen, orbital ultrasonography and magnetic resonance imaging (MRI). The patient’s clinical activity score (CAS) was determined, and thyroid function tests, antithyroid antibody levels, and B lymphocytes were evaluated. Chest radiograph, routine biochemistry, liver function tests, hepatitis screening (prophylaxis based on results), and immunoglobulin levels were measured to screen for potential side effects of rituximab. The patient’s pretreatment visual acuity was 0.8 in the right eye and 0.9 in the left eye. There was bilateral eyelid edema which was more pronounced on the right, and eyelid retraction was evident. The palpebral aperture was 16 mm on the right and 13 mm on the left. The conjunctivae were hyperemic, and chemosis and caruncular edema were apparent in the right eye. Eye movement in the right eye was limited in upgaze. There was pronounced proptosis bilaterally: 26 mm on the right and 23 mm on the left with a base measure of 105 mm. Orbital MRI revealed bilateral thickening of the medial rectus (MR; 6.25 mm right, 4.8 mm left) and inferior rectus (IR; 8.1 mm right, 7.2 mm left) muscles. Hess screen test revealed underaction of the left IR muscle. The patient reported spontaneous pain in the right eye and her CAS was 7/7 in the right and and 5/7 in the left eye. Intraocular pressure in the right and left eye was 23 mmHg and 22 mmHg in primary gaze position and 27 and 24 mmHg in upgaze, respectively. Visual field and VEP tests were normal. Antithyroid antibody levels were elevated (antithyroglobulin antibody: 717.2 IU/mL, TSHR antibody: 20.98 U/L); thyroid hormones and B lymphocytes were within normal range. Systemic screening prior to rituximab therapy revealed no pathologies. The patient received 2 infusions of 1000 mg IV rituximab administered 2 weeks apart. To prevent allergic reaction, 1 g paracetamol and 10 mg chlorpheniramine were administered prior to infusion. After the second dose of rituximab was administered, improvements were observed in the soft tissue findings of eyelid edema, hyperemia, conjunctival edema, hyperemia, and caruncular edema. CAS score was 5/7 for the right and 4/7 for the left eye. Orbital MRI revealed significant reduction in MR (5.6 mm right, 4.6 mm left) and IR (5.6 mm right, 5.5 mm left) muscle thickness, but there was no change in the degree of proptosis. Antithyroid antibody levels decreased to baseline levels (antithyroglobulin antibody: 606.5 IU/mL, TSHR antibody: 13.56 U/L). There was also improvement in the patient’s signs of psoriasis. The patient has been followed for 4 months and no treatment-related side effects have been observed. Figures 1, 2 and 3 show pre- and posttreatment images of the patient’s eyes, orbital MRI, and psoriatic lesions.

## DISCUSSION

GO is an autoimmune disease resulting from cross-reactivity between antigens of the thyroid and orbital tissues. Stimulation of orbital TSHRs induces the release of glycosaminoglycans from fibroblasts, which in turn alters the osmotic balance, leading to fluid retention and an increase in orbital volume.^9,10^

Although GO is usually mild and resolves spontaneously, it may be severe enough to threaten sight in 3-5% of cases.^9^ The course of the disease has two distinct stages, an active phase followed by an inactive phase. Signs and symptoms in the active phase include proptosis, lid hyperemia, periorbital edema, conjunctival hyperemia (particularly near the extraocular muscles), chemosis, caruncular edema, diplopia, corneal ulceration, and rarely vision loss due to optic nerve compression.^3^ These findings are associated with inflammation, glycosaminoglycan accumulation, and increased adipose tissue. As the bony orbit cannot expand, an increase in orbital volume disrupts venous drainage, leading to edema and chemosis in the orbital region.^3,9^ The active phase is marked by the effect of cytokines (interleukin-6, interleukin-1, and gamma interferon) released from T helper (Th) type 1 cells.^10,11^

The inactive phase is characterized by more stable proptosis, lid retraction, and restrictive strabismus, whereas signs of inflammation resolve. The disease generally enters the inactive phase an average of 18-24 months after onset.^3^ The cytokines involved in this phase originate from T helper-2 (Th2) cells and are predominantly interleukin-4, 5 and 10.^11^

In addition to T cells, B cells also play a role in the pathogenesis of GO. It has been demonstrated that B cells are abundant in the orbits of GO patients and that they produce autoantibodies against TSHR and IGF-1R. Aside from producing antibodies, B cells present antigens to T cells and mediate their activation via cytokines.^12^ Therefore, agents that are effective against both T cells and B cells are now used in the management of GO.^13^

Currently, the most common therapeutic approaches to GO are corticosteroids, radiotherapy, and decompression surgery.^13,14^ Long-term corticosteroid use can lead to hypertension, hyperglycemia, diabetes, osteoporosis, cushingoid appearance, proximal myopathy, peptic ulceration, increased susceptibility to infections, and psychiatric disorders.^15^ External-beam radiotherapy can cause temporary exacerbation of ocular symptoms and cataract. Furthermore, it is not generally recommended for diabetic and hypertensive patients due to the risk of radiation retinopathy, a rare but sight-threatening complication. It is also not recommended for patients under 35 years of age because it can precipitate carcinogenesis.^16,17^ Orbital decompression surgery is performed in the active phase when there is a threat to optic disc function, or for cosmetic purposes in the inactive phase. The procedure requires an experienced surgical team.^13,14,18^ Due to the limitations of these therapeutic modalities, the search for effective treatment for GO is ongoing. The efficacy of rituximab in other autoimmune diseases such as rheumatoid arthritis suggested that it may also be effective in the management of GO.

Rituximab is a monoclonal antibody against the transmembrane protein CD20, which is expressed in both mature and immature B cells. The CD20 antigen enables the activation and differentiation of B cells. As CD20 is absent in plasma and stem cells, rituximab can prevent B cell activation and differentiation without disrupting the immunoglobulin structure or B cell regeneration. In GO, which primarily involves T cells, rituximab also disrupts B cells’ antigen-presenting function.^19,20^ Rituximab has been shown to deplete B cells for 4-6 months and reduce signs associated with active GO. The earliest study related to this was presented by Salvi et al.,^6^ who reported an extremely rapid (within hours) regression of proptosis and clinical signs after rituximab treatment.

To date, most relevant studies are case reports or small case series; no controlled clinical studies have been conducted yet. Khanna et al.^21^ administered rituximab to 6 patients whose active GO was unresponsive to steroid therapy and orbital decompression surgery. All of the patients showed improved CAS and reduced orbital inflammation, but proptosis and strabismus remained unchanged. The authors concluded that rituximab does not offer a cure for GO but can be considered as a salvage therapy, particularly in cases of serious complications like optic neuropathy. Similarly, our case did not show improvement in proptosis or eye movement restriction despite regression of signs related to soft tissue involvement. The presence of another autoimmune disease, normal baseline B lymphocyte levels, or having previously underwent 8 cycles of adalimumab therapy for psoriasis may explain why our patient did not exhibit rapid improvement following rituximab treatment. Furthermore, patients in other studies of rituximab were administered combined therapy with corticosteroids. We did not use that approach due to our patient’s contraindication for steroid use. In a study evaluating patients treated with rituximab, it was noted that 2 of the 3 patients who did not respond to treatment had received only rituximab, whereas all of the responsive patients had been treated with both rituximab and corticosteroids. Combined use of the two agents may have provided a quicker effect and greater improvement of symptoms.^22^ A case showing transient improvement after rituximab therapy with later recurrence has also been reported.^23^ Based on our experience with the present case, we believe that rituximab treatment was effective against some factors involved in the pathogenesis of GO, but the disease still showed progression due to other pathogenetic mechanisms.

GO is an autoimmune disease and may manifest with other autoimmune diseases, the most common of which are rheumatoid arthritis and type 2 diabetes. Comorbid dermatologic disorders such as pemphigus vulgaris and acquired ichthyiosis have also been reported.^24^ A search of the literature yielded no other cases like our own, with GO and coexisting psoriasis. The Th1 inflammatory cytokines involved in the active phase of GO are also responsible for the pathogenesis of psoriasis.^25^ The effect of rituximab on psoriasis is controversial. There are reports of patients developing psoriasis after rituximab therapy, but there are also cases whose cutaneous lesions and psoriatic arthritis partially improved after taking rituximab.^26,27^ Interleukin-10-secreting regulatory B cells mediate the suppression of autoimmune and inflammatory diseases by inhibiting Th1 and Th2 cytokine polarization, antigen presentation, and proinflammatory cytokine production by monocytes and macrophages. B cell depletion after treatment with drugs like rituximab may result in exacerbation of autoimmune diseases like ulcerative colitis and psoriasis.^28,29^ The proposed mechanism by which rituximab causes psoriasis is that the depletion of B cells eliminates their regulatory effect over T cells, resulting in an abnormal T cell response or subclinical infection which triggers psoriasis.^26^ Rituximab treatment has also been reported to bring about partial amelioration of psoriatic lesions, but those patients were under rituximab therapy for lymphoma or other hematologic diseases and had coexisting psoriasis. The improvement of psoriatic plaques in these patients has been associated with the effect of rituximab on immune complexes that mediate the production of tumor necrosis factor alpha (TNF-α).^27^ TNF-α blockers are used in the management of psoriasis.

Our patient was also administered the TNF-α blocker adalimumab for psoriasis prior to rituximab therapy. After rituximab, she exhibited no increase in psoriatic lesions and in fact a partial improvement was observed. This may be attributed to the effect of rituximab on TNF-α. Our patient exhibited no side effects related to rituximab therapy during the 4 months of follow-up. Potential short-term side effects such as hypotension, sinus tachycardia, and serum sickness have been reported in the literature. Rare instances of polyarthritis, ulcerative colitis, urinary system infections, cardiac arrest and pneumonia have been reported in the long-term.^22^ The low incidence of serious side effects makes rituximab therapy advantageous.

Smoking is another important issue in the management of GO, and patients who smoke are strongly advised to quit. Cigarette use is proven to increase the prevalence and severity of GO. Moreover, smoking is a cause of poor treatment response. Smoking has been shown to increase the synthesis of hyaluronic acid by orbital fibroblasts in adipose tissue; furthermore, tissue hypoxia induced by smoking results in the generation of free radicals, which in turn induce the proliferation of orbital fibroblasts and stimulate glycosaminoglycan synthesis and adipogenesis.^30,31^ Our patient also had a smoking history. We believe this had a negative impact on her recovery and resulted in only partial resolution of her symptoms.

Although thyroid function is not associated with GO severity, the treatment of hyperthyroidism is undeniably important. GO is more severe in the presence of persistent hyper- or hypothyroidism; therefore, it is recommended to maintain a state of euthyroidism in these patients. Due to higher incidence and severity of GO with radioactive iodine therapy, this treatment is not recommended for high-risk patients.^3^

## CONCLUSION

Although GO is rarely severe, its management is still of substantial importance due to possible sight-threatening complications and resultant esthetic issues and reduced quality of life. Because of the many factors involved in the pathogenesis, a single therapeutic approach may not be effective and different treatment protocols may be necessary. In our case, IV rituximab therapy prevented serious complications by arresting disease progression and effecting partial resolution of symptoms, indicating that IV rituximab is a possible alternative therapy for patients with contraindication for steroids or other treatment approaches. Randomized, controlled clinical studies are needed to evaluate the efficacy and safety of this treatment method.

### Ethics

Informed Consent: It was taken.

Peer-review: Externally and internally peer-reviewed.
